# Recurrent Inflammatory Flares in HIV-Infected Patients: Consider Castleman Disease!

**DOI:** 10.1177/2324709617725094

**Published:** 2017-09-08

**Authors:** Sabrina Jegerlehner, Andri Rauch, Urban Novak

**Affiliations:** 1 Inselspital, Bern University Hospital, University of Bern, Bern, Switzerland

**Keywords:** inflammatory flares, Castleman disease, HIV infection, treatment, rituximab, etoposide

## Abstract

*Background*. Transient inflammatory flares are common in clinical care of human immunodeficiency virus (HIV)–infected patients. In-depth investigations are not performed routinely because patients often recover without therapeutic interventions, and therefore, the etiologies of these inflammatory flares frequently remain unknown. *Case*. We report a case of an HIV-infected patient with recurrent inflammatory flares during several years in whom diagnostic workup with a lymph node biopsy finally revealed multicentric Castleman disease (MCD). The patient was treated with etoposide and rituximab until November 2013 and achieved ongoing complete clinical remission. *Conclusion*. Recent effective therapeutic regimens offer an opportunity to prevent serious complications of MCD including its malignant transformation, provided that the diagnosis is established early enough. Therefore, clinicians should consider MCD in the differential diagnosis of self-limiting inflammatory flares, especially in HIV-infected patients.

## Introduction

Transient inflammatory flares are common in clinical care of human immunodeficiency virus (HIV)–infected patients, even in the setting of a well-controlled infection. However, the etiologies of theses flares often remain unknown due to recovery without therapeutic interventions, lack of in-depth investigation, or misdiagnosis—with potential deleterious consequences. We report a case of an HIV-infected patient with recurrent inflammatory flares in whom broader diagnostic workup finally revealed multicentric Castleman disease (MCD), and therefore could be treated successfully with achievement of ongoing clinical remission.

## Case Presentation

**Figure 1.**
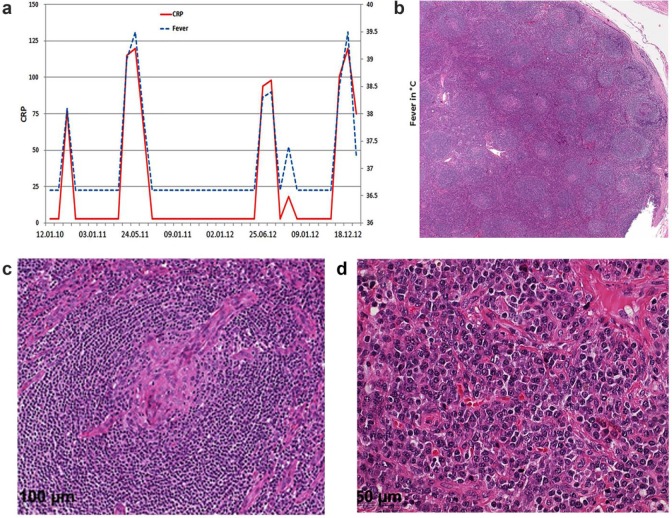
(a) Course of fever and CRP (C-reactive protein) over time during the repeated inflammatory flares. (b-d) Lymph node biopsy with the characteristically expanded mantle zone with abundant interfollicular plasma cells and a radially penetrating sclerotic blood vessel (“lollipop” sign).

A 38-year old male from Cameroon was diagnosed with HIV-1 infection in 2003. His initial CD4 T cell count was 408/µL and the HIV viral load 67 700 copies/mL. In February 2004, he presented with a cutaneous Kaposi sarcoma on the right foot. Antiretroviral therapy (ART) was started in 2008, and was continued to date without treatment interruptions. HIV-RNA was always below the limit of detection until the last follow-up visit and CD4 T cell counts increase from a nadir of 258 cells/µL to a maximum of 758 cells/µL. The cutaneous Kaposi lesions regressed after ART initiation and local radiation therapy. Since March 2010, the patient suffered from recurrent inflammatory flares with high fever, myalgia, arthralgia, and nasal congestion (Figure 1a). These bouts were accompanied by normocytic normochromic anemia followed by thrombocytosis and elevated inflammatory markers. Initially, these episodes responded well to anti-inflammatory drugs with defervescence, normalization of C-reactive protein (CRP) values and thrombocyte counts, as well as complete regression of lymphadenopathy and myalgia, and therefore were interpreted as inflammatory reactions to viral pathogens. A more severe flare with significant lymphadenopathy, splenomegaly, and profound anemia (hemoglobin of 75 g/L) initiated a broader diagnostic workup in December 2012. Radiologic imaging with computed tomography scan demonstrated generalized lymphadenopathy and hepatosplenomegaly. Repeated blood cultures remained sterile, and workup for viral hepatitis, Epstein-Barr virus, cytomegalovirus, herpes simplex, or respiratory viral infection was negative. There was no evidence of endocrine, rheumatological, or autoimmune disorder. A lymph node biopsy revealed diagnostic scattered onionskin fibrosis of germinal centers and prominent interfollicular plasmocytosis (Figure 1b-d). However, immunoglobulin heavy chain (IgH) gene rearrangement studies ruled out a clonal disorder. Polymerase chain reaction analysis was positive for human herpes virus type 8 (HHV-8) in both the lymph node biopsy and in plasma. The HHV-8 viral load in plasma was 54 200 copies/mL during the severe inflammatory flare, but undetectable in between. These features along with the clinical presentation confirmed the diagnosis of the plasma cell variant of MCD. Treatment was initiated with 1-cycle etoposide induction and 4 weekly rituximab, followed by 4 administrations of rituximab every other month as consolidation until November 2013. This led to a rapid resolution of all symptoms, diminution of lymphadenopathy, and normalization of CRP and anemia. Kaposi sarcoma scars regressed and paled. Until the last clinical visit in September 2016, about 30 months after initiation of the chemotherapy, there was complete clinical remission without further inflammatory flares.

## Discussion

Castleman disease is a nonclonal lymphoproliferative disorder. Localized manifestation is differentiated from the multicentric form typically observed in HIV-infected patients. Histopathologically, hyaline vascular variant is discerned from plasma cell variant and from the more recently described plasmablastic subtype.^1,2^ MCD is a systemic disease, sometimes associated with HHV-8 and other malignancies such as Kaposi sarcoma, and is histologically characterized by the plasma cell variant.^3-5^ The plasmablasts can coalesce to form microlymphoma and can progress to plasmablastic monoclonal lymphoma.^6,7^ The “lollipop” sign is a characteristic feature formed by radially penetrating sclerotic blood vessel. HHV-8 encodes viral interleukin-6 (IL-6) and induces expression of the pro-inflammatory cytokine IL-6 in infected B cells. In contrast to the incidence of Kaposi sarcoma, the incidence of MCD has increased in the ART era, further indicating that immune dysregulation rather than immunosuppression is the underlying pathophysiological mechanism of HIV-related MCD.^8^

The clinical criteria to define MCD were described by the CastlemaB Trial Group,^9^ and include fever, raised serum CRP (>20 mg/L) in the absence of another cause, and 3 of 12 additional clinical findings (lymphadenopathy, splenomegaly, edema, pleural effusion, ascites, cough, nasal obstruction, xerostomia, rash, neurologic symptoms, jaundice, autoimmune hemolytic anemia). However, these unspecific symptoms render the diagnosis of Castleman disease difficult based on clinical symptoms alone. As a result, the diagnosis and therefore therapy are often delayed.

Therapy for MCD in HIV-infected patients is a combination of ART, chemotherapy, and rituximab, with the goal to achieve fast disease control. Studies using rituximab reported an overall survival of up to 95% with disease-free survival of up to 79%.^9,10^

Because the inflammatory flares in our patient initially regressed with nonsteroidal anti-inflammatory drug treatment, elaborate diagnostics was not deemed needed and the diagnosis of Castleman disease was missed for more than 2 years. However, after diagnosis by lymph node biopsy and initiation of therapy, he achieved an ongoing clinical remission of 30 months so far.

## Conclusion

This case demonstrates that clinicians should consider MCD in the differential diagnosis of self-limiting inflammatory flares in HIV-positive patients, even in the setting of an immunologically and virologically well-controlled HIV infection. Recent effective therapeutic regimens offer an opportunity to prevent the serious complications of MCD including its malignant transformation,^11^ provided that the diagnosis is established early enough.

## References

[1] van Rhee F Stone K Szmania S Barlogie B Singh Z Castleman disease in the 21st century: an update on diagnosis, assessment, and therapy. Clin Adv Hematol Oncol. 2010;8:486-498. 20864917

[2] Saeed-Abdul-Rahman I Al-Amri AM Castleman disease. Korean J Hematol. 2012;47:163-177. 23071471 10.5045/kjh.2012.47.3.163 PMC3464333

[3] Flending JA Shillings PHM Benign giant lymphoma: the clinical signs and symptoms. Folia Med Neerl. 1996;12:119.

[4] Keller AR Hochholzer L Castleman B Hyaline-vascular and plasma-cell types of giant lymphnode hyperplasia of the mediastinum and other locations. Cancer. 1972;29:670-683. 4551306 10.1002/1097-0142(197203)29:3<670::aid-cncr2820290321>3.0.co;2-#

[5] Oksenhendler E HIV-associated multicentric Castleman disease. Curr Opin HIV AIDS. 2009;4:16-21. 19343828 10.1097/coh.0b013e328319bca9

[6] Li CF Ye H Liu H Du MQ Chuang SS Fatal HHV-8-associated hemophagocytic syndrome in an HIV-negative immunocompetent patient with plasmablastic variant of multicentric Castleman disease (plasmablastic microlymphoma). Am J Surg Pathol. 2006;30:123-127. 16330952 10.1097/01.pas.0000172293.59785.b4

[7] Dupin N Diss TL Kellam P et al HHV-8 is associated with a plasmablastic variant of Castleman disease that is linked to HHV-8-positive plasmablastic lymphoma. Blood. 2000;95:1406-1412. 10666218

[8] Casper C The aetiology and management of Castleman disease at 50 years: translating pathophysiology to patient care. Br J Haemotol. 2005;129:3-17. 10.1111/j.1365-2141.2004.05311.x 15801951

[9] Gérard L Bérezné A Galicier L et al Prospective study of rituximab in chemotherapy-dependent human immunodeficiency virus associated multicentric Castleman’s disease: ANRS 117 CastlemaB Trial. J Clin Oncol. 2007;25:3350-3356. 17664482 10.1200/JCO.2007.10.6732

[10] Bower M Powles T Williams S et al Brief communication: rituximab in HIV-associated multicentric Castleman disease. Ann Intern Med. 2007;147:836-839. 18087054 10.7326/0003-4819-147-12-200712180-00003

[11] Gérard L Michot JM Burcheri S et al Rituximab decreases the risk of lymphoma in patients with HIV-associated multicentric Castleman disease. Blood. 2012;119:2228-2233. 22223822 10.1182/blood-2011-08-376012

